# Complete genome sequence of deformed wing virus and black queen cell virus isolated from honeybees (*Apis mellifera*) in Argentina

**DOI:** 10.1128/mra.01025-23

**Published:** 2024-02-01

**Authors:** Fernanda N. Gonzalez, Cecilia Ferrufino, María José Dus Santos, Hugo A. Carignano

**Affiliations:** 1 Instituto de Virología e Innovaciones Tecnológicas, Instituto Nacional de Tecnología Agropecuaria-Consejo Nacional de Investigaciones Científicas y Técnicas, Hurlingham, Argentina; 2 Consejo Nacional de Investigaciones Científicas y Técnicas, Ciudad Autónoma de Buenos Aires, Argentina; DOE Joint Genome Institute, Berkeley, California, USA

**Keywords:** deformed wing virus, black queen cell virus, complete genome sequence, *Apis mellifera*

## Abstract

We report the complete genome sequence of deformed wing virus and black queen cell virus isolated from Argentinean’s honeybees. These sequence data will be valuable for future research on the viral variants present in the country and the development of strategies to control the spread of these viruses in apiaries.

## ANNOUNCEMENT

Bee viruses contribute significantly to honey production losses and are associated with elevated morbidity and mortality in both naïve and wild bee populations (1, 2). Furthermore, the decline of honeybees poses a threat to our food production system, given their pivotal role in providing pollination services to crops (3). The black queen cell virus (BQCV) and deformed wing virus (DWV) (order *Picornavirales*, families *Dicistroviridae* and *Iflaviridae*, respectively) are among the most commonly encountered viruses in colonies (4).

Genomic sequences of DWV and BQCV have been reported worldwide (taxid:198112 and taxid:92395, respectively), providing essential resources to study the epidemiology, pathogenesis, and control strategies of these viruses. However, in South America, only a DWV complete genome from Chile has been described (5). In this study, we present the complete genome sequences of DWV and BQCV obtained from *Apis mellifera* workers gathered in the Entre Rios province, Argentina, in September 2019.

Viral particles from a DWV-BQCV doubled positive sample, identified by Reverse transcription-quantitative polymerase chain reaction (RT-qPCR) as in references (6) and (7), were purified by a sucrose gradient ultracentrifugation method (8) and *in vitro* propagated (9). Bee pupae were inoculated with 25 µL of viral particles and incubated [(30°C and 60% Relative humidity (RH)] for 3 days. Then, the pupae were macerated and centrifugated (4,500 rpm at 4°C for 45 min). The supernatant was washed twice with chloroform, applied to a sucrose gradient (15%–41.25%), and ultracentrifuged (28,000 rpm at 4°C for 3 hours). Total RNA was extracted from the purified virus using the TIANamp Virus RNA kit (Tiangen Biotech, China). A 100-ng aliquot was prepared using the TruSeq RNA Kit-v2 (Illumina, US) and subsequently sequenced on a NovaSeq 6000 platform, producing 71,023,737 reads (2 × 150 bp). Raw reads were subjected to quality trimming using BBMap v35.85 (BBMap-sourceforge.net/projects/bbmap/), discarding reads shorter than 50 nt.

Clean reads were *de novo* assembled using rnaSPAdes v3.15.5 (10). Two contigs identified as complete viral genomes by the Viralverify and Viralcomplete modules (11) underwent a BLASTn search against the NCBI nonredundant database (12); the best alignments scores revealed a sequence identity of 97.42% with DWV (accession no. AY292384.1) and 96.17% with BQCV (accession no. MH267693.1). Open reading frames (ORFs) and functional protein domains were annotated using Prokka v1.14.5 (13) and InterProScan v.5.55-88.0 (14), respectively.

The DWV-Argentina-ER308 genome was 10,187 nt in length (coverage 128,709, %GC = 37.80), whereas BQCV-Argentina-ER308 had 8,566 nt (coverage 48,044, %GC = 40.30).

The DWV-Argentina-ER308 has a genome organization consisting of a single ORF (position: 1,132–9,813 nt, 2,893 aa) encoding a polyprotein flanked by 5′ and 3′ UTRs and a poly(A) tail.

The BQCV-Argentina-ER308 genome aligns with the *Dicistroviridae* family, featuring a 5′-end ORF1 (690–5,576 nt, 1,628 aa) and a 3′-end ORF2 (5,892–8,345 nt, 817 aa) for the non-structural and structural polyproteins, respectively. The ORF domain signatures for both viruses can be accessed at DWV-ORF, BQCV-ORF1, and BQCV-ORF2.

**Fig 1:**
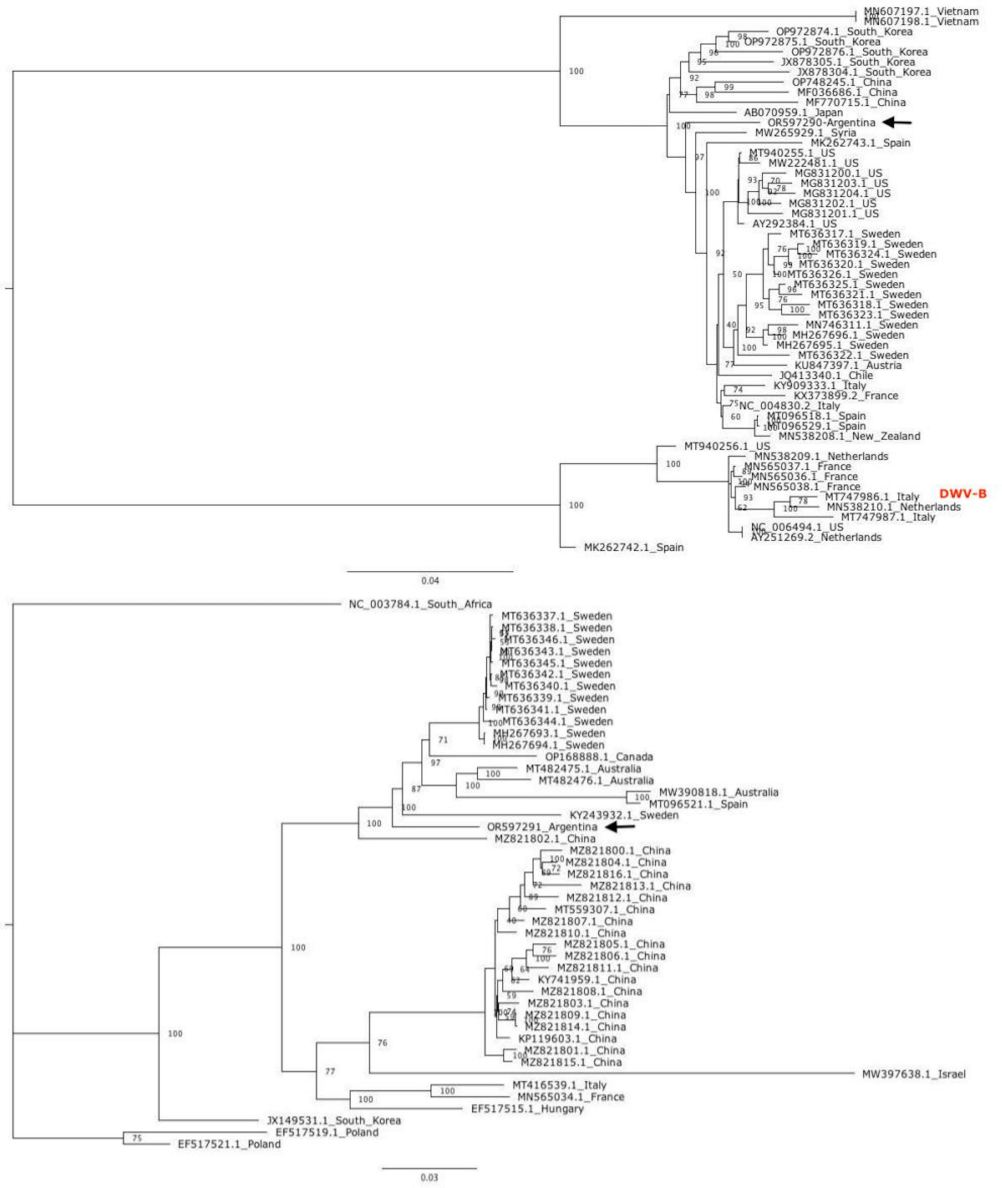
Maximum-likelihood phylogenetic trees of DWV-Argentina-ER308 (**A**) and BQCV-Argentina-ER308 (B). Phylogenetic trees were constructed with the complete genome sequences available for DWV (GenBank taxid:198112) and BQCV (GenBank taxid:92395), using IQ-TREE v.1.6.12 (15) with default parameters. The evolutionary models were automatically selected with ModelFinder (16), and branch confidence was determined using the Ultrafast bootstrap approximation with 1,000 replicates (17). Bootstrap values are indicated near nodes. Black arrows point to DWV-Argentina-ER308 and BQCV-Argentina-ER308. In A, the tree was re-rooted on the DWV-B clade; all others DWV genomes correspond to A-variant. DWV-C recombinant complete genome sequences were excluded from the analysis.

DWV-Argentina-ER308 is related to DWV-A genotypes of Asiatic origin (Fig. 1A), while BQCV-Argentina-ER308 is most closely associated with European isolates (Fig. 1B).

## Data Availability

The complete genome sequences of DWV-Argentina-ER308 and BQCV-Argentina-ER308 were deposited in GenBank under the accession numbers OR597290 and OR597291, respectively. Raw sequencing reads are available in the NCBI Sequence Read Archive (SRA) under the accession number SRR26222246 (BioSample: SAMN37524832).
